# Roles of the CCR4‐Not complex in translation and dynamics of co‐translation events

**DOI:** 10.1002/wrna.1827

**Published:** 2023-11-27

**Authors:** Martine A. Collart, Léna Audebert, Martin Bushell

**Affiliations:** ^1^ Department of Microbiology and Molecular Medicine Institute of Genetics and Genomics Geneva, University of Geneva, Faculty of Medicine Genève Switzerland; ^2^ Cancer Research UK Beatson Institute Glasgow UK; ^3^ School of Cancer Sciences, University of Glasgow Glasgow UK

**Keywords:** Ccr4‐Not, codon optimality, Not5 ribosome interaction, repression of translation initiation, translation elongation dynamics

## Abstract

The Ccr4‐Not complex is a global regulator of mRNA metabolism in eukaryotic cells that is most well‐known to repress gene expression. Delivery of the complex to mRNAs through a multitude of distinct mechanisms accelerates their decay, yet Ccr4‐Not also plays an important role in co‐translational processes, such as co‐translational association of proteins and delivery of translating mRNAs to organelles. The recent structure of Not5 interacting with the translated ribosome has brought to light that embedded information within the codon sequence can be monitored by recruitment of the Ccr4‐Not complex to elongating ribosomes. Thereby, the Ccr4‐Not complex is empowered with regulatory decisions determining the fate of proteins being synthesized and their encoding mRNAs. This review will focus on the roles of the complex in translation and dynamics of co‐translation events.

This article is categorized under:

Translation > Mechanisms
Translation > Regulation

## Graphical abstract

Not5 ribosome binding ensures co‐translational organellar targeting and protein assembly. Prolonged ribosome pausing leading to post‐translocation ribosomes with empty A and E sites allows Not5 ribosome binding. This Not5 ribosome binding is key for co‐translational events such as organellar targeting or assembly of proteins.

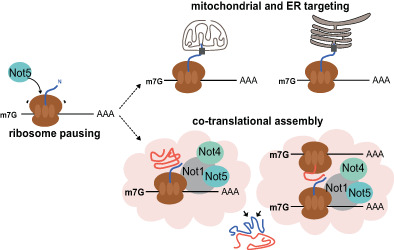

## 1 INTRODUCTION

The Ccr4‐Not complex is a conserved multi‐subunit L‐shaped complex that regulates mRNA metabolism at all stages, from production of the mRNA in the nucleus to its decay in the cytoplasm. An ever‐increasing plethora of physiological functions that critically depend upon Ccr4‐Not are being discovered, as are diseases associated with mutations in this complex (De Keersmaecker et al., 2013; Faraji et al., 2014; Faraji et al., 2016; Wang et al., 2017; Wong et al., 2015). This review will focus on the roles of the complex in translation and dynamics of co‐translation events that have increasingly come into the spotlight in the last couple of years since a structure of the Not5/CNOT3 subunit (yeast/human name) interacting with the translating ribosome has been described (Buschauer et al., 2020).

**FIGURE 1:**
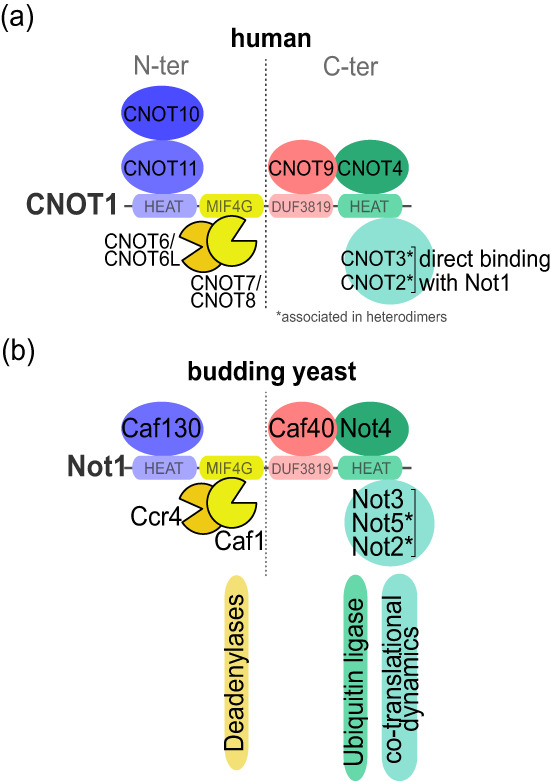
Cartoon representation of the human (a) and budding yeast (b) Ccr4‐Not complexes. The human complex has alternative deadenylase subunits such that 4 complexes of different composition can be formed depending upon which deadenylase subunits are present.

The Ccr4‐Not complex is conserved across the eukaryotic kingdom, though there are some differences in composition (Figure 1a,b) (Arae et al., 2019; Bui et al., 2016; Hart et al., 2019; Raisch & Valkov, 2022). It is built upon a central scaffold protein Not1/CNOT1, onto which the other subunits corresponding to functional modules assemble, providing Not1/CNOT1 with immense regulatory capacity. This includes two enzymatic modules. The first is a deadenylase module composed of Ccr4/CNOT6 and Caf1/CNOT7 that bind a central MIF4G domain of Not1/CNOT1 (Basquin et al., 2012; Petit et al., 2012; Zhang et al., 2022). The second is a ubiquitination module composed of the Not4/CNOT4 RING E3 ligase, that docks onto a C‐terminal region of Not1, just upstream of the site where a Not5‐Not2/CNOT3‐CNOT2 heterodimer docks (Bhaskar et al., 2015; Temme et al., 2010; E. Wu et al., 2017). In metazoans and flies Not4/CNOT4 is a non‐constitutive subunit of the Ccr4‐Not complex. It has a conserved mode of interaction with the complex via the Caf40/CNOT9 subunit that, however, deviates substantially from its direct interaction with Not1 in yeast and is of low affinity (Keskeny et al., 2019). In mammals another E3 ligase, RNF219, co‐purifies with the CCR4‐NOT complex (Du et al., 2020; Guenole et al., 2022) via the CNOT9 module (Poetz et al., 2021). Caf40/CNOT9 docks onto a DUF3819 domain of Not1/CNOT1, C‐terminal to the MIF4G domain (Y. Chen et al., 2014). In yeast there is a subunit, Not3, with homology to Not5, particularly in its N‐terminus, that associates with all other Ccr4‐Not subunits (Collart & Struhl, 1994). Caf130 is a core subunit of the yeast Ccr4‐Not complex without known ortholog in human (J. Chen et al., 2001), that associates with the very N‐terminus of Not1 (amino acids 21–153) (Pillet et al., 2022). In flies and metazoans instead, a CNOT10‐CNOT11 heterodimer associates with an N‐terminal domain of CNOT1 (Mauxion et al., 2013; Mauxion et al., 2023). In addition to the proteins considered as bona fide Ccr4‐Not subunits, other proteins that interact with the Not1 scaffold have been identified. Some of these are RNA binding proteins that serve to recruit the Ccr4‐Not complex to target mRNAs such as for instance Tristetraprolin (TTP) (Fabian et al., 2013; Sandler et al., 2011), or GW182 of the miRNA‐induced silencing complex (miRISC) required for miRNA‐mediated gene silencing (Fabian et al., 2011). Other proteins such as Dhh1/DDX6 (Mathys et al., 2014; Raisch et al., 2018; Rouya et al., 2014) or eIF4A2 (Meijer et al., 2013; Meijer et al., 2019; Wilczynska et al., 2019) function through the MIF4G domain of CNOT1 and are likely effectors of Ccr4‐Not function as will be described below.

## 2 EARLY EVIDENCE FOR A ROLE OF CCR4‐NOT IN TRANSLATION

Mutations in the *NOT* genes were isolated in a selection for increased expression of the *HIS3* gene and a *HIS3*‐lacZ reporter in budding yeast in 1993, 1994 and 1998 (Collart & Struhl, 1993, 1994; Oberholzer & Collart, 1998). It was noted that mutations in the *NOT* genes increased expression of the mRNA produced from the TATA‐less promoter of the *HIS3* gene, but not of the mRNA transcribed from the TATA‐box‐dependent promoter. At the time of these observations, there was no understanding that translation and stability of 2 nearly identical transcripts could be different, and the critical importance of co‐translational decay for mRNA turnover emerged only later in 2009 (Hu et al., 2009). Indeed, it was thought that the presence of ribosomes on mRNAs prevented their decay, until it was determined that decapped mRNAs were associated with ribosomes (Hu et al., 2009). Instead, at the time of the isolation of the *not* mutations, cDNAs encoding TBP had just been isolated (Hernandez, 1993) and differences in transcription from promoters with or without a TATA box were being characterized (Yang et al., 2007). Hence, it was concluded that the *not* mutations impacted transcription.

In 1999 came the discovery that the Not proteins were in a complex with Ccr4 and Caf1 (Bai et al., 1999), and in 2001, Ccr4 and Caf1 were characterized as deadenylating enzymes (Daugeron et al., 2001; Tucker et al., 2001). Even though there was compelling evidence that the Not proteins were functionally different from Ccr4 and Caf1 (Bai et al., 1999), this drove a major focus of research on the Ccr4‐Not complex as a deadenylase complex, and in 2006 it was shown that recruitment of Caf1 to a target mRNA induced its rapid degradation (Finoux & Seraphin, 2006). In the following years, the importance of the poly(A) tail length to modulate gene expression became evident, and this topic was covered by several reviews (Beilharz & Preiss, 2007; Goldstrohm & Wickens, 2008). On one hand deadenylation was described as the rate limiting step in the major pathway for mRNA turnover in eukaryotes, and on the other many studies described the role of poly(A) tails in promoting translation initiation (Beilharz & Preiss, 2007; Kapp & Lorsch, 2004). Hence, the widespread understanding less than 10 years after the first description of the Ccr4‐Not complex was that regulated removal of the poly(A) tail by recruitment of the Ccr4‐Not complex to its target mRNAs, repressed gene expression and determined mRNA fate (Wiederhold & Passmore, 2010). Many studies investigated whether repression of translation was an indirect result of deadenylation. Instead, evidence for mechanisms of translation repression independent of deadenylation mediated by Ccr4‐Not recruitment to mRNAs in different organisms arose in the early days of Ccr4‐Not biology (Cooke et al., 2010; Jeske et al., 2006; Van Etten et al., 2012). In addition, the advent of several methods to evaluate globally poly(A) tail lengths such as PAL‐Seq (Subtelny et al., 2014), mTAIL‐Seq (Lim et al., 2016) and nanopore sequencing (Tudek et al., 2021; Workman et al., 2019) have called into question the connection of poly(A) tail length to translation and mRNA stability. Some studies are proposing that long poly(A) tails are not systematically correlated with high translation efficiency, but rather that the presence of the poly(A) tail‐binding protein Pab1/PABC1 is the determining factor (Lima et al., 2017; Xiang & Bartel, 2021) and a recent study has shown that deadenylation and degradation are actually uncoupled during meiosis in yeast cells (Wiener et al., 2021).

In budding yeast, it was immediately noted that deletion of the Not proteins was more detrimental to cell growth than deletion of the deadenylase subunits (Azzouz et al., 2009). It was also observed that mutations in the *NOT* genes, or their deletion, resulted in aggregation of newly synthesized proteins, and that this was not observed in cells lacking the deadenylase subunits (Halter et al., 2014). Moreover, mutations in ribosomal protein genes or in genes encoding ribosome biogenesis factors were identified in the same selection as the *NOT* genes (see above) (Collart et al., 2017) whereas, mutations in *CCR4* or in the *CAF* genes did not pass this selection (Halter et al., 2014). These were all hints for functional heterogeneity of the Ccr4‐Not complex and suggestions that the *NOT1‐5* genes may have been identified in a selection revealing factors defective in the translation process. Not4 was then characterized as a RING E3 ubiquitin ligase in 2001 (Hanzawa et al., 2001). In 2006, its first substrate in budding yeast was identified as a ribosome‐associated chaperone, namely the Nascent polypeptide Associated Complex (NAC), composed of Egd1, Btt1 and Egd2 (Panasenko et al., 2006). These combined observations lay the foundation to investigate a possible role of the Not proteins in translation in budding yeast. They did not immediately have a major impact on the overwhelming majority of studies on Ccr4‐Not biology that were in mammals and flies, because Not4/CNOT4 is not a stable subunit of the complex in those organisms. However, in 2019 the mode of interaction of Not4/CNOT4 with the Ccr4‐Not complex in higher eukaryotes was finally described (Keskeny et al., 2019) indicating that Not4/CNOT4 was most certainly generally relevant for Ccr4‐Not function.

## 3 TRANSLATION

While the Ccr4‐Not complex plays a major role in mRNA stability determination, here we will focus on the evidence that has been accumulating around its function in the process of protein synthesis which has been accumulating over the last 15 years. In the following sections we will summarize our current knowledge of the relevance of the Ccr4‐Not complex at each stage of the translation process, namely initiation, elongation, termination, and ribosome recycling from multiple organisms.

## 4 TRANSLATION INITIATION

**FIGURE 2:**
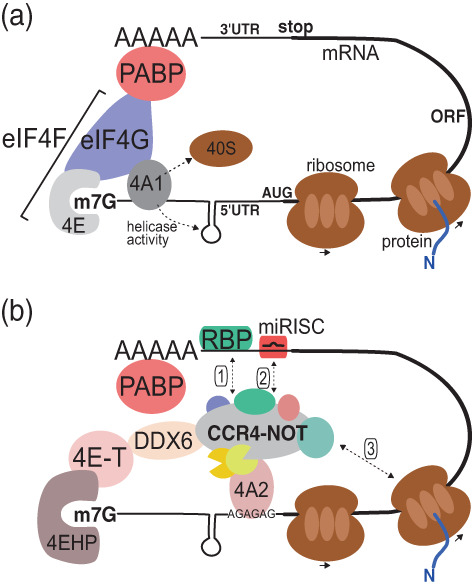
Cartoon representation of the interactions of the Ccr4‐Not complex impacting translation initiation. (a) Closed loop translation initiation complex. (b) Recruitment of the human Ccr4‐Not complex to mRNAs by either RNA binding proteins (RBPs, 1), the microRNA machinery (miRISC, 2) or the translating ribosome (3) to repress initiation via DDX6, 4E‐T, 4EHP or eIF4A2 interactions. Notably, some mRNAs may have limited association of their 3′ and 5′ends, how this impacts communication between these elements is currently unclear and needs further investigation (Vicens et al., 2018).

Translation initiation is the first step in protein synthesis, during which the small subunit of the ribosome, with the initiator methionyl‐tRNA, is loaded onto the mRNA and then scans the 5′ untranslated region (5′UTR) of an mRNA to identify an AUG through recognition by the initiator tRNA. Key to these events is the function of the cap‐binding complex, eIF4F, which consists of the cap binding protein, eIF4E, a large regulatory central platform for complex formation, eIF4G, and the catalytic subunit eIF4A1. Importantly, eIF4A has been shown to have two critical roles in translation initiation, first, it loads mRNA onto the small ribosomal subunit and second through its helicase function unwinds RNA allowing the ribosome to scan the 5′UTR for the start codon (Kumar et al., 2016; Shirokikh et al., 2019; Sokabe & Fraser, 2017; Svitkin et al., 2001; Yourik et al., 2017). The eIF4F complex also associates through the eIF4G subunit with the poly(A) binding protein, which in turn is associated with mRNA poly(A) tails, and this circularization of the mRNA is thought to contribute to regulate translation initiation (Figure 2a). Therefore, it is easy to imagine that deadenylating enzymes can regulate translation initiation through this circularized configuration. However, as mentioned above, evidence has quickly emerged that translation could be repressed by Ccr4‐Not independently of deadenylation, showing that this form of repression relied on cap‐dependent mRNA translation (Cooke et al., 2010). Perhaps unsurprisingly then, the vast majority of the mechanisms proposed to mediate this repression at initiation center are around the inhibition of the eIF4F complex (Figure 2b).

Early observation of the association of the Ccr4‐Not complex with the mRNA decapping apparatus paved the way for one of the major mechanisms through which the Ccr4‐Not complex led to translation repression (Tucker et al., 2002). A number of these factors interact with the Ccr4‐Not subunits and in turn, interact with and repress, the function of components of the translation initiation machinery, mainly eIF4E (the cap binding subunit). This results in deactivation of the eIF4F complex and dismantlement of the mRNA closed loop structure. One such factor is 4E‐T (eIF4E transporter). It interacts with LSM14, the LSM1‐7‐PAT1 complex and eIF4E, in a manner which is mutually exclusively to eIF4G binding to eIF4E (Nishimura et al., 2015). Another factor is 4EHP (cap binding eIF4E‐homologous binding protein). It competes with eIF4E binding to 4E‐T, and when associated with 4E‐T, has more affinity for the Cap, forming an alternative closed loop that blocks translation initiation (Chapat et al., 2017).

The central component of the Ccr4‐Not complex, CNOT1 has several domains containing HEAT repeats with similarities to domains found within eIF4G. Of particular interest is one of the central HEAT repeats which resembles the eIF4A binding site within eIF4G, called the MIF4G domain. This domain is found in a number of proteins and acts as a site for RNA helicase interactions (Mathys et al., 2014). These observations together with data showing that a stage downstream of cap recognition (Kamenska et al., 2014; Meijer et al., 2013) was being targeted, initiated a search for possible helicases that could interact with this site on Not1/CNOT1. Two helicases have been identified to interact with this central MIF4G domain of CNOT1: eIF4A2 (whereas eIF4A1, a close paralogue, is associated with eIF4G) and DDX6 (ortholog of yeast Dhh1) (Y. Chen et al., 2014; Mathys et al., 2014; Rouya et al., 2014; Waghray et al., 2015), with DDX6 also known to interact with 4E‐T (Minshall et al., 2007). Interestingly, eIF4A1 is a well‐known translational activator as part of the eIF4F cap binding complex, whereas eIF4A2 appears to be associated with mRNAs that are translationally repressed (Wilczynska et al., 2019). Both eIF4A2 and DDX6 have been proposed to repress translation by a Cap‐ and eIF4E‐independent mechanism, needing eIF4G (the central hub protein) and eIF4A (helicase) or eIF4B (Kamenska et al., 2014; Meijer et al., 2013). Interestingly, this particular domain within CNOT1 also houses the binding site for CNOT7, on the opposite surface to the helicase interaction site (Y. Chen et al., 2014; Mathys et al., 2014).

Subsequent analysis of the roles of these helicases in controlling mRNA translation has highlighted that the majority of the mRNAs associated with either of the helicases are associated with both helicases; however, there are a number of mRNAs more specially bound with one of the two helicases. Exploring the features of mRNAs associated specifically only with one of the helicases highlights certain features, including the lack of enrichment for miRNAs target sites within the DDX6 only associated mRNAs. mRNAs associated with eIF4A2 reside more in sub‐polysomes (monosomes and disomes) and are enriched for miRNA target sites of some miRNA families, while the mRNAs associated with both eIF4A2 and DDX6 are enriched in miRNA target sites that are mainly distinct from those found in eIF4A2 bound mRNAs (Wilczynska et al., 2019). Moreover, while DDX6 bound mRNAs are more associated with polysomes in control conditions, following CNOT1 knockdown, DDX6‐bound mRNAs shift into the sub‐polysomal fraction and are not up‐regulated. This is very distinct from the eIF4A2‐bound mRNAs which shift to the polysome fractions, consistent with mRNAs released from repression at initiation. Proteomic analysis showed consistently that the mRNAs bound by eIF4A2 had increased protein production following CNOT1 depletion. However, surprisingly DDX6 bound mRNAs showed less increases in protein production than the unbound majority of mRNAs (Wilczynska et al., 2019). Further examination of how eIF4A2 is functioning to repress translation at initiation on these mRNAs showed that it is associating with GA motifs within these mRNAs towards the 5′ends and inhibiting translation initiation. These data together suggested that binding of eIF4A2 was inhibiting translation by directing initiation to upstream AUG start sites (Wilczynska et al., 2019). How mechanistically DDX6 functions in this is still unclear, but the changes in polysome association of mRNAs bound by DDX6 appear to suggest a role in regulating protein synthesis post‐initiation, when the ribosome is translocating along the mRNAs during decoding of the polypeptide chain. In budding yeast, the homologue Dhh1 has been implicated in linking reduced ribosome translocation due to stalled elongation to mRNA decay (Radhakrishnan et al., 2016). Interestingly, the differential motifs within the mRNAs bound by eIF4A2 and DDX6 suggest that different mechanisms of Ccr4‐Not complex recruitment might be involved. While eIF4A2‐bound mRNAs contain miRNA target sites, there is a lack of an enrichment of miRNA target sites in DDX6 only‐bound mRNAs (Wilczynska et al., 2019). This could suggest that these mRNAs recruit DDX6 through direct sensing of codon optimality, which would be expected to induce ribosome stalling, as will be discussed later.

The close proximity of the helicase binding site within the MIF4G domain of CNOT1 with the binding site of CNOT7, one of the deadenylation subunits within the CCR4‐NOT complex, suggests potential communication between these components. Interestingly, using purified components, it is clear that association of DDX6 with the MIF4G domain and CNOT7 activates the deadenylation capacity of CNOT7, while eIF4A2 inhibits it (Meijer et al., 2019). Together, these observations suggest that the helicases are deployed on mRNAs to direct distinct outcomes, with DDX6 triggering mRNA decay and regulation post‐initiation, while eIF4A2 directs translation repression at initiation but limits the ability of Ccr4‐Not complex to deadenylate the mRNAs.

It should be stated that others have questioned if eIF4A2 is involved in miRNA‐mediated regulation and whether this stage of translation is targeted by miRNAs (Galicia‐Vazquez et al., 2015; Kuzuoglu‐Ozturk et al., 2016). The recent developments highlighted in this review have shown that the Ccr4‐Not complex can function at multiple levels and thus redundancy within the repression system may be occurring, as has been shown previously (Meijer et al., 2019). Additionally, with the realization that codon usage is being monitored by the Ccr4‐Not complex (Buschauer et al., 2020), this new variable should be evaluated in these different experimental conditions.

Recently a number of helicases have been shown to play critical roles in phase separation, including eIF4A1 (Tauber et al., 2020). Whether eIF4A2 also operates at this level is currently unclear; however, the mRNAs associated with eIF4A2 are enriched for mRNAs found in processing bodies (P‐bodies) (Wilczynska et al., 2019). In budding yeast, Dhh1 associated with mRNAs can phase separate into liquid droplets that are dissolved by Not1 (Mugler et al., 2016). The roles of phase separation in mRNA storage, translation decay and translational repression are only beginning to be explored, hence how these helicases contribute to these processes needs to be examined in greater depth.

In budding yeast, fewer studies have associated Ccr4‐Not with regulation of translation initiation, and mechanistic insight is rather limited. Ccr4 has been proposed to contribute to translational repression in stationary phase cells (Duy et al., 2017), but this might indirectly be due to the role of Ccr4 for down‐regulation of ribosome biogenesis and ribosome mRNAs during the shift from glucose to glucose‐depleted medium (Grigull et al., 2004). Not4 also contributes to translational repression after glucose or amino acid starvation, decreasing amino acid incorporation and polysome formation (Preissler et al., 2015). In this context, it is interesting to note that a target of Not4 ubiquitination, the ribosomal protein Rps7A (see below), must be de‐ubiquitinated for effective translation re‐initiation (Ikeuchi et al., 2023; Takehara et al., 2021). The absence of any Rps7A ubiquitination might be related to the reduced translation repression observed after starvation, when cells lack Not4. Such a model can easily be tested. It is interesting to note that in cells lacking Not4 or Not5, ribosomes accumulate massively at the start codon (Allen et al., 2021), and that Not4 and Not5 associate with ribosomes at the start codon (Buschauer et al., 2020), suggesting a role of these proteins at translation initiation, or at the switch from translation initiation to translation elongation.

## 5 TRANSLATION ELONGATION

Translation elongation represents the step during which the ribosome translates the genetic code and synthesizes the encoded protein. The dynamics of translation elongation are determined by many factors including codon composition, they are specific for each transcript and are not constant throughout a transcript. Codon optimality is a measure that reflects the balance between the supply of charged tRNA molecules in the cytoplasmic pool and the demand for tRNA usage by the translating ribosomes, itself dependent upon the transcriptome. Codon optimality provides a measure of translation efficiency per‐codon. There are a number of different suggestions as to how this can be calculated as discussed in (Gillen, Waldron, & Bushell, 2021). Many events accompany the process of translation elongation, such as modifications and folding of the nascent chain, and interaction of the nascent chain with chaperones or partner proteins. Moreover, both co‐translational quality control responses and initiation of mRNA decay can occur during the translation elongation phase. Not5 in association with Not4 can interact with the translating ribosome and modulate translation elongation dynamics according to codon optimality. As will be summarized below, the association of Not4 and Not5 with the elongating ribosome is necessary for the presence of chaperones or partner subunits of the nascent chain at the site of translation, as well as for their co‐translational interaction. Moreover, Not4 contributes to co‐translational quality control responses limiting protein overexpression that endangers cellular protein homeostasis. These findings support the roles of Not4 and Not5 during elongation.

### 5.1 Co‐translational association of proteins

**FIGURE 3:**
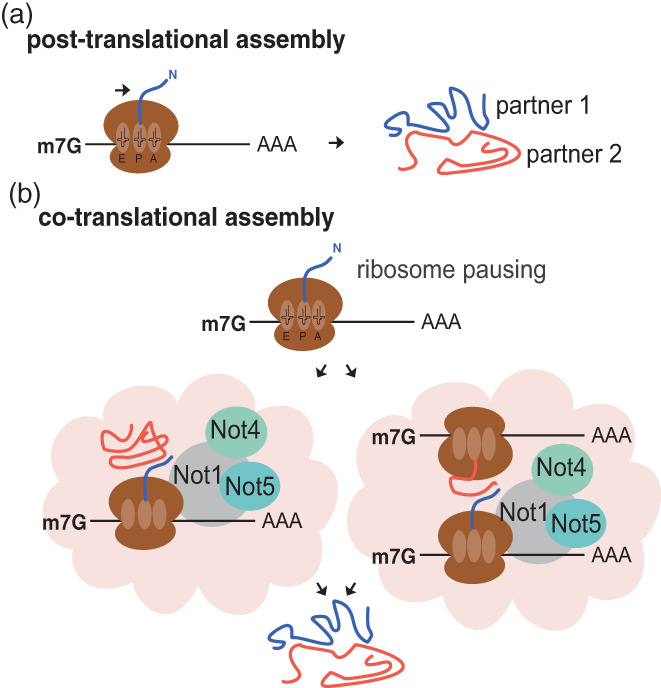
(a). Proteins can assemble post‐translationally. Assembly occurs when synthesis of each partner has been completed. (b). Proteins can assemble co‐translationally. During translation protein domains fold with the help of chaperones or assemble with partner proteins co‐translationally. This process is helped by modulation of translation dynamics, ribosome pausing, and recruitment of Not proteins to the mRNA being translated, helping to recruit chaperones or partner proteins fully synthesized (left), or helping to co‐localize 2 mRNAs translating partner proteins (right).

Most cellular proteins do not work alone, but assemble with other proteins to exert their cellular function, and some functions are mediated by assemblies of a large number of different proteins. We still have only minimal knowledge about how complexes assemble in the cell, but in the last 10–15 years, this question has been raising increasing interest. Assembly is not only post‐translational (Figure 3a), but also co‐translational (Figure 3b), and principles of co‐translational assembly are emerging (Khan & Fox, 2023; Kramer et al., 2019; Morales‐Polanco et al., 2022; Shiber et al., 2018).

The first hints for a role of the Not proteins in assembly of protein complexes came from 2 observations in budding yeast. First, the comparison of total protein extracts from wild type cells and cells lacking individual Not proteins showed no differences by SDS‐PAGE but important differences by Native‐PAGE (Kassem et al., 2017; Panasenko & Collart, 2011; Villanyi et al., 2014). Second, some but not all, subunits of multiprotein complexes, co‐purified with the Not proteins (Kassem et al., 2017). This idea was investigated for several different well characterized protein complexes and obtained experimental support as will be outlined below.

RNA polymerase II (RNAPII) is a nuclear complex composed of 12 subunits. The 2 largest subunits, Rpb1 and Rpb2, form in the cytoplasm assembly‐competent intermediate complexes comprised of the specific RNAPII subunits and dedicated chaperones, before coming together to form the mature RNAPII complex that can be imported into the nucleus following the release of the chaperones (Wild & Cramer, 2012). Rpb1, is an aggregation prone protein, and its interaction with its dedicated chaperone (R2TP‐Hsp90) during translation is necessary for the formation of the soluble and assembly‐competent intermediate complex. In the absence of Not5, Rpb1 aggregates and the Rpb2 assembly intermediate complex lacking Rpb1 accumulates. The co‐translational protection of Rpb1 is thought to be mediated by Not5‐dependent association of Not1 with the *RPB1* mRNA, itself required for recruitment of the Rvb2 co‐chaperone to the site of translation of Rpb1 (Villanyi et al., 2014).

The SAGA histone acetyltransferase complex is a nuclear complex composed of 19 subunits in yeast and organized in functional modules. The structural organization of SAGA has been very well characterized and summarized in several recent reviews (Ben‐Shem et al., 2021; Cheon et al., 2020; Elias‐Villalobos et al., 2019) but we still know very little about how this complex is assembled in yeast. For mammalian cells, it has been recently demonstrated that co‐translational assembly plays an important role (Kamenova et al., 2019). The mRNAs encoding Ada2 of the HAT module co‐localizes with the mRNAs encoding Gcn5 and Spt20 of the Hat and Spt modules, respectively, in yeast. In the absence of Not5, Gcn5 complexes are compromised, and Ada2, Gcn5 and Spt20 accumulate in speckles in the cytoplasm rather than localize in the nucleus where the integral SAGA complex resides. The interaction of Not1 with the *ADA2* mRNA and the presence of the Ada2 protein at the site of Spt20 synthesis depends upon Not5. Not4 is also present at the site of Spt20 synthesis, as is Tdh3 (glyceraldeyde‐3‐phosphate‐dehydrogenase), that moonlights as a chaperone for SAGA assembly. Indeed, in its absence, as in the absence of Not4 or Not5, Gcn5 accumulates in cytoplasmic speckles. This co‐translational assembly of SAGA subunits is thought to rely upon Not5‐dependent association of Not1 with the *ADA2* mRNA (Kassem et al., 2017).

The third example is the proteasome, formed by the assembly of 2 core and 2 regulatory particles, and composed of over 26 subunits. Two of the proteasome base subunits, Rpt1 and Rpt2, are synthesized with ribosome pausing to allow mRNA co‐localization and co‐translational association of the nascent chains in Not1 granules, named Not1‐containing assemblysomes (NCAs). This is conserved from yeast to human (Panasenko et al., 2019) and correlates with the importance of Not4 for the assembly of the proteasome base (Panasenko & Collart, 2011). In addition, Not4 competes with specific base chaperones to ubiquitinate Rpt5 and block premature incorporation of lid subunits (Fu et al., 2018).

All of these examples describe the critical role played by the recruitment of Not1, by Not4 and/or Not5‐dependent mechanisms, to the site of translation, for chaperone delivery or co‐translational assembly of partner subunits (Figure 3b). In turn this is important for assembly of functional and integral multi‐subunit protein complexes. It appears that the N‐terminal domain of Not1/CNOT1 is important for docking of factors that interact with nascent chains, via Caf130 in yeast (Pillet et al., 2022) and CNOT10/CNOT11 in mammalian cells (Hopfler et al., 2023). While the general theme emerging from these studies is the same, at the mechanistic level, very little has been decrypted. How is Not1 brought to the site of translation? How do the partner proteins and/or mRNAs co‐localize? How does the presence of Not1 enable the co‐translational interactions? When is Not1 recruited, when does it dissociate? Most of these questions still do not have answers, but some mechanistic advances have nevertheless been achieved.

### 5.2 Interaction of the Not proteins with the translating ribosome

The presence of Not proteins in polysome fractions, corresponding to ribosomes engaged in translation elongation, was first described many years ago in budding yeast (Dimitrova et al., 2009; Panasenko & Collart, 2012). In those early days of Ccr4‐Not discovery, the ubiquitination of NAC by Not4 was shown to improve its ribosome association (Azzouz et al., 2009). Moreover, a ribosomal subunit, Rps7A, was also identified as a substrate for Not4, and the ubiquitinated form of Rps7A was detected in polysome fractions, but not in free 40S ribosomes (Panasenko & Collart, 2012). The ubiquitination of Rps7A by Not4 was also shown to increase the presence of Not5 in polysome fractions (Panasenko & Collart, 2012). These findings hinted to Not4 being able to interact with translating ribosomes to ubiquitinate Rps7A and NAC, a model also supported by the co‐purification of all ribosome subunits with the Not proteins (Panasenko et al., 2019), although other models could explain those observations. These first experiments did not distinguish whether the Not proteins interacted directly with the ribosome, with mRNAs being translated or with proteins recruited to nascent chains.

This issue was finally clarified in 2020 when the Beckmann laboratory obtained a structure of the yeast Not5 N‐terminal domain in the ribosomal E site of post‐translocation ribosomes with empty A sites after purification of Not4‐associated ribosomes (Buschauer et al., 2020). Ribosomes with an empty A site are more likely to be observed if the A site is occupied by a non‐optimal codon, and profiling of Not4‐associated ribosomes revealed an enrichment of ribosomes with a non‐optimal codon in the A site, as well as initiating ribosomes with the start codon in the P‐site (Buschauer et al., 2020). Interestingly, ribosome profiling comparing cells lacking Not5 with wild type cells, revealed increased ribosomes with non‐optimal A‐site codons (called A‐site ribosome dwelling occupancy [A‐site RDO]), with A‐site RDO changes inversely correlating with codon optimality (Allen et al., 2021). In other words, an increase in translating ribosomes able to be bound by Not4 and Not5 in wild type cells was observed in the absence of Not5.

Curiously, codon‐specific changes in A‐site RDOs in *not4Δ* do not show any correlation with those in *not5Δ* (Allen et al., 2021), though the structural work suggests that Not4 and Not5 together are associated with post‐translocation ribosomes (Buschauer et al., 2020). Ribosome profiling provides information on the dwelling of all translating ribosomes. Instead, profiling of 5′P decay intermediates (5′P‐Seq) provides information on dwelling of the last translating ribosome (Pelechano et al., 2015). Indeed, the A site of ribosomes that prevent progression of the Xrn1 exonuclease is located 17 nucleotides downstream of the 5′P‐nucleotide of the decay intermediates. One can hence map ribosome dwelling and determine whether A site ribosome dwelling shows any codon bias. Notably, the dynamics evaluated using 5′P‐Seq are those of the last translating ribosome and concern decapped mRNAs. Using 5′P‐Seq data, A‐site RDO changes in *not4Δ* and *not5Δ* compared to wild type were nearly identical (Allen et al., 2021), showing again increased A‐site RDOs at non‐optimal codons, with an overall inverse correlation between A‐site RDO changes and codon optimality.

This suggests that the structure of ribosomes associated with Not4 and Not5 obtained by Beckmann and collaborators is compatible with the last translating ribosomes that accumulate and limit progression of the Xrn1 exonuclease, in the absence of Not4 and Not5, but also after Not1 depletion, since codon‐specific 5′P‐Seq A‐site RDO changes correlate with those in *not4Δ* and *not5Δ* (Allen et al., 2021; Allen et al., 2023). It may seem contradictory that the Not proteins are contributing to co‐translational assembly if they are associated with decay intermediates whose abundance increase in their absence. However, this could reflect their role in quality control of co‐translational assembly as will be discussed further (see below). The Beckman study also revealed that the Not proteins interact with ribosomes at start, namely ribosomes with the start codon in the P site. Ribo‐Seq indicates that ribosomes with the start codon in the P‐site accumulate in the absence of Not4 or Not5, or in other words, ribosomes dwell more at start. Hence, again ribosomes with which the Not proteins associate in wild type cells accumulate in the absence of the Not proteins. In this case also it seems counterintuitive that Not proteins bind to the ribosome to degrade mRNAs that have not yet been translated (see discussion below).

### 5.3 mRNA solubility and translation elongation dynamics

**FIGURE 4:**
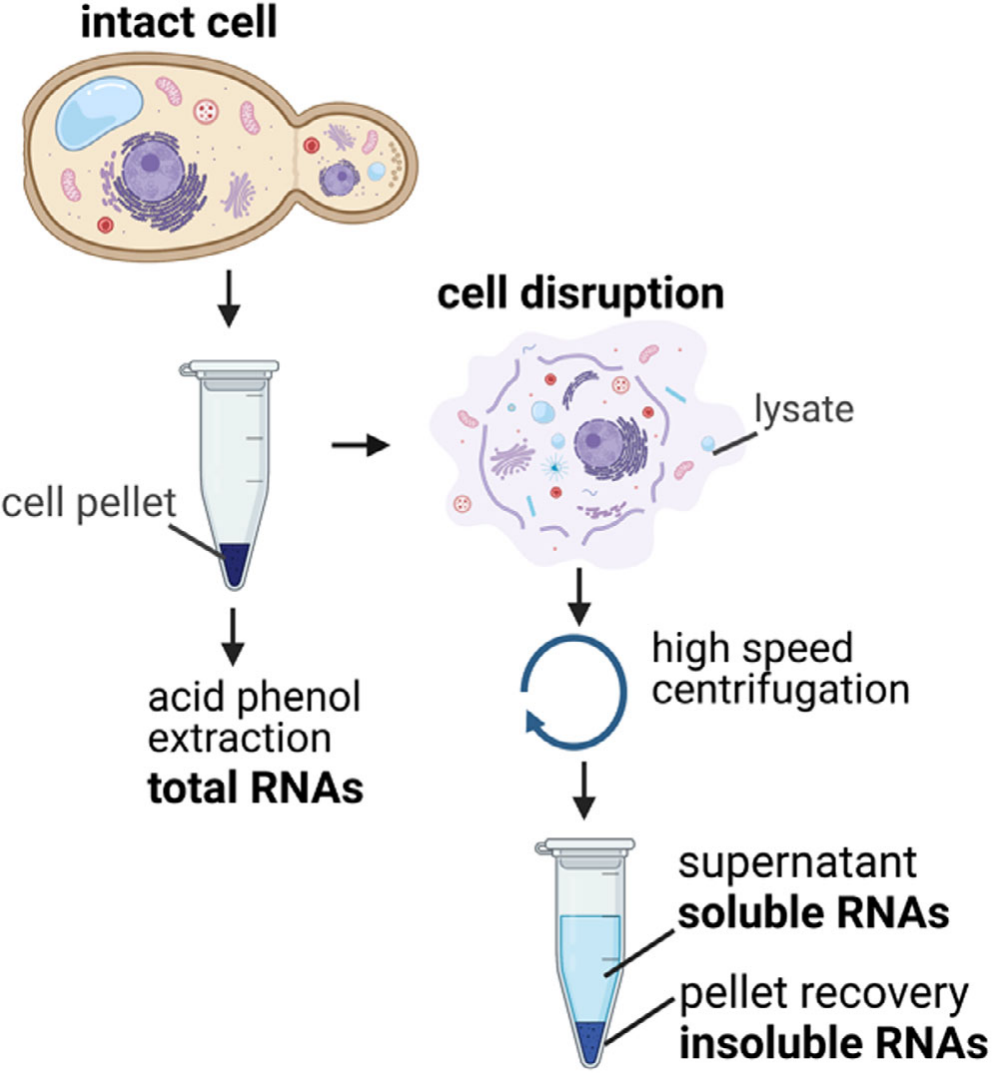
Extraction of soluble and total mRNAs. To extract total RNAs, intact cells are pelleted and total RNAs are commonly prepared by acid phenol extraction or by phenol‐containing commercial kits with chaotropic agents. To prepare soluble RNAs, intact cells are lysed by freeze–thaw cycles, mechanical or liquid membrane disruption in presence of salt and non‐ionic detergent, followed by high‐speed centrifugation. The soluble RNAs are extracted from the supernatant. The pellet contains cellular debris (e.g., membranes, cell wall) and any attached RNAs (insoluble RNA pool).

To get a better understanding of what may be the role of Not proteins recruited to ribosomes in dynamics of co‐translation events, we need to consider the main tools available to gain mechanistic insights, namely Ribo‐Seq and 5′P‐Seq. Ribo‐Seq data provides information on the position of ribosomes on mRNAs that are being translated and are present in soluble cellular extracts recovered after separation from membranes and other heavy cellular debris. Ribosomes on mRNAs potentially associated with membranes and heavy cellular debris are hence excluded. 5′P‐Seq data provides information on decapped mRNAs that are being degraded, and it can be obtained for the entire cellular RNA pool, but can also be obtained specifically for the same pool of soluble mRNAs as Ribo‐Seq. 5′P‐Seq data detects intermediates of post‐translational and co‐translational decay and it is able to provide information on the dynamics of the last translating ribosome. Indeed, it has been noted that 5′P‐decay intermediates show a 3‐nucleotide periodicity indicating that the Xrn1 exonuclease follows the last translating ribosome (Pelechano et al., 2016) as mentioned above. This was confirmed by an observation in cryo‐EM showing a physical interaction between Xrn1 and the *Saccharomyces cerevisiae* 80S (Tesina et al., 2019). Additionally, what we have learned about the interaction of Not proteins with the ribosome comes from purification of Not4‐associated complexes that uses as a starting material soluble cellular extracts (Buschauer et al., 2020). We should also keep in mind that the content of soluble cellular extracts can be variable depending upon which conditions were used to prepare such extracts, for instance which conditions of salt or detergent (Shaiken et al., 2023) (Figure 4).

In recent work it was shown preparing cell extracts under conditions of usual polysome or ribosome profiling (50 mM KCl or 150 mM NaCl, 0.1% Triton X‐100) and preparing total RNA by extracting the RNA directly from cell pellets with acid phenol, that the solubility of mRNAs is diverse and that translation elongation dynamics of mRNAs was different depending on the extraction method (Allen et al., 2023). Indeed, it was noted that ribosome dwelling with non‐optimal A‐site codons was higher for insoluble mRNAs. Moreover, ribosomes dwell longer at non‐optimal codons in the second half of coding sequences than in the first half, but particularly for insoluble mRNAs and much less for soluble mRNAs. Whether differences in solubility of mRNAs is an intrinsic property of the mRNAs or whether they are associated with the translation properties of the mRNAs remains to be clarified. There is some initial evidence that both the nature of the mRNA and its translation can contribute (see below). In addition, a recent study has indicated that differential translation in viscous fluid and solid elastic compartments, named cytosol and cytomatrix, reflects a structural organization of functional networks (Shaiken et al., 2023).

In budding yeast 5′P‐Seq has been used to analyze translation elongation dynamics of both the soluble and total RNA pools (including the insoluble RNAs) (Allen et al., 2023). From these studies it was noted that changes in A‐site RDOs occur according to codon optimality upon depletion of Not proteins in the soluble RNA pool (by Ribo‐Seq and 5′P‐Seq) and that this is mediated by a specific group of mRNAs whose solubility (distribution between soluble and total RNA pools) changes. An exciting observation has been that Not1 and Not4 had opposite effects: Not1 is important for solubility of mRNAs that are well expressed and enriched for optimal codons, whereas Not4 is important for solubility of mRNAs with less optimal codons. In turn, 5′P‐Seq of soluble RNAs revealed that upon depletion of Not1, A‐site RDOs at non‐optimal codons increases, whereas inversely upon depletion of Not4: A‐site RDOs at non‐optimal codons decreases. Instead, upon depletion of Not proteins there is no codon‐optimality related changes in A‐site RDOs detectable for the total RNA pool, hence no overall change in translation elongation dynamics according to codon‐optimality. This is strong evidence to indicate that the primary impact of the Not proteins might be to modulate solubility of specific categories of mRNAs and in turn impact detectable translation elongation dynamics according to codon optimality in the soluble RNA pool. Notably, the dynamics revealed in these studies are those of the last translating ribosome. Detectable here refers to analyses such as Ribo‐Seq, but maybe in cells also soluble mRNAs are differently accessible to factors such as those involved in mRNA turnover compared to insoluble mRNAs.

The roles of Not4 and Not5 have also been studied by Ribo‐Seq, comparing wild type cells with cells lacking Not4 or Not5 (Allen et al., 2021). These studies concern the soluble RNA pool and the dynamics of all translating ribosomes. The deletion of Not5 resulted in increased A‐site RDOs at non‐optimal codons overall, correlating with A‐site RDO changes detected by 5′P‐Seq in *not5Δ* or upon Not1 depletion. They also nicely correlate with changes in production of new proteins detected by SILAC in *not5Δ*. Curiously, these changes inversely correlate with changes detected by 5′P‐Seq upon Not5 or Not4 depletion. This suggests that at steady state, cells lacking Not5 may behave as cells with limiting Not1 with regard to translation elongation dynamics, and the ribosomes in the soluble RNA pool reflect nicely the ribosomes that are actively translating. Instead, immediately upon depletion of Not5 or upon depletion of Not4 (within 15 min) the consequence is the same, which is maybe that the proteins do not associate with the ribosome to counteract the impact of Not1. Another observation is that in the absence of Not4, A‐site RDO changes detected by Ribo‐Seq do not show any correlation to codon optimality. They also do not correlate with changes in new protein production. Hence, in the absence of Not4, the pool of ribosomes in the soluble RNA pool do not reflect appropriately the actively translating ribosomes. This observation is compatible with an important role of Not4 in regulation of the solubility of actively translating ribosomes (Allen et al., 2021).

Notably, in human cells the formation of P‐bodies where mRNA decay is reported to take place, has been described to be reduced upon CNOT1 depletion (Ito et al., 2011) and AU‐rich mRNAs are enriched in P‐bodies indicating that codon bias is associated with P‐bodies (Courel et al., 2019; Hubstenberger et al., 2017). CNOT1 condensates are distinct from P‐bodies in human cancer cell lines (Panasenko et al., 2019) and in *Caenorhabditis elegans* mRNA degradation components and CCR4‐NOT complexes form distinct foci (Daskalaki et al., 2023). Nevertheless, one can speculate that there could be a cross‐talk between P‐bodies and CNOT1 condensates that regulate solubility of mRNAs and translation elongation dynamics.

### 5.4 Role of the Not proteins

The study from the Beckman laboratory (Buschauer et al., 2020) showed that an artificial mRNA with extreme non‐optimal codon content is more unstable than one with an extreme high optimal codon content confirming an earlier study from the Coller laboratory (Presnyak et al., 2015), and that this is dependent upon Not5. Previously it was reported to depend upon Dhh1 (Radhakrishnan et al., 2016). Beckmann and colleagues concluded that Not4 and Not5 monitor the translating ribosomes for turnover of mRNAs according to codon optimality. This is reminiscent of previous studies indicating that Ccr4‐Not mediated deadenylation and mRNA degradation are connected to codon optimality (Presnyak et al., 2015; Webster et al., 2018). However, while these observations seem easily compatible with a role of Not5 in degradation of the mRNA according to codon optimality, no mechanism for degradation has been identified and maybe this straightforward interpretation is not the right one, especially considering that it appears contradictory with the role of the Not proteins for co‐translational assembly of protein complexes. Indeed, the detection of increased mRNA decay intermediates in the absence of Not proteins does not necessarily mean that the Not proteins are usually responsible for their turnover. An alternative explanation could be that the presence of the Not proteins hinders the detection of the decay intermediates by RNA condensation: both ribosome profiling and purification of Not4‐associated complexes use as input starting material soluble cellular extracts.

**FIGURE 5:**
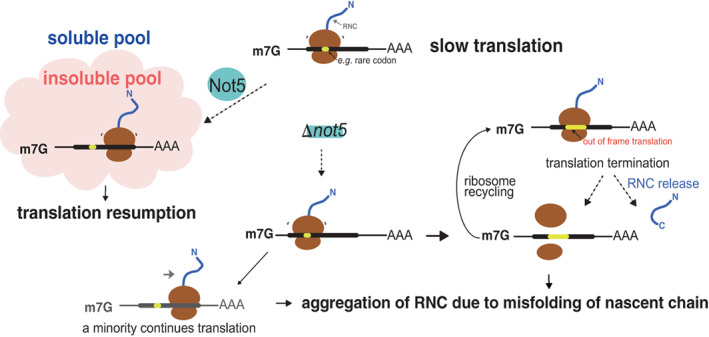
Hypotheses to explain protein aggregation and translation elongation defects in cells lacking Not5. When post‐translocation ribosomes pause long lastingly when both E and A sites are empty, Not5 can bind the ribosome E site and initiate condensation of the ribosome nascent chain (RNC) complex. In condensates translation resumes but translation dynamics are different and nascent chain folding and interactions can occur (left panel). In the absence of Not5 the RNC remains soluble. Because the elongation dynamics are hence not appropriate, or chaperones and partners of the nascent chain cannot interact with the nascent chain, this can provoke aggregation of the RNC (bottom middle panel) or frameshifting followed by translation elongation until a stop codon in the inappropriate frame followed by translation termination (bottom right panel). Consequently, the amount of translation that will proceed normally through the coding sequence (bottom left panel) is reduced compared to wild type cells.

Let us imagine for instance that binding of Not5 tethers ribosome nascent chain complexes (RNCs) to condensates to promote interactions in condensates or avoid interactions in the cytosol, and maybe momentarily slows translation to enable co‐translational events, as has been proposed (Figure 5). Indeed, eIF5A, the translation initiation/elongation factor is absent in Not5 condensates (Allen et al., 2021). Thus, in the absence of Not5, ribosomes that are normally bound by Not5 are likely to be translated with inappropriate dynamics. This could result in aggregation of the RNC. This scenario is compatible with more ribosome footprints at the beginning of coding sequences and less at the end for the soluble RNA pool that is observed specifically in *not5Δ* (Allen et al., 2021) as well as with new protein aggregation in *not5Δ* (Panasenko & Collart, 2012). Another mechanism that would be compatible with these observations would be that the absence of Not5 impacts reading fidelity after prolonged dwelling at start, leading to frameshifting, followed by out of frame translation termination. Indeed, as mentioned above, accumulation of ribosome footprints was observed at start in *not5Δ* (Allen et al., 2021) and Not5 can associate with ribosomes at start (Buschauer et al., 2020). This model can easily be tested. Since the A‐site RDO changes defined by Ribo‐Seq and 5′P‐Seq correlate very specifically for *not5Δ*, this would indicate that in *not5Δ* codon‐specific effects detected are being devised from the last translating ribosomes. Both proposed mechanisms, translation that continues until RNC aggregation or frameshifting and subsequent translation termination, would end translation regardless of the specific codon in the A site. Compatible with this is the observation that in cells lacking Not5, changes in A‐site RDOs measured by 5′P‐Seq for the soluble and total RNA pools correlate (Allen et al., 2021). This suggests that the insoluble mRNAs contribute minimally if at all to codon‐specific ribosome dwelling.

A common point in cells lacking Not4 or Not5 is that newly synthesized peptides/proteins aggregate (Halter et al., 2014; Panasenko & Collart, 2012). However, one difference in *not4Δ* compared to *not5Δ* is that there is no translation elongation defect (Allen et al., 2021). The presence of Not5 in *not4Δ* appears to be sufficient to prevent an elongation defect but not protein aggregation. Maybe ribosomes in *not4Δ* produce full‐length proteins with inappropriate dynamics that aggregate. Alternatively, in *not4Δ*, RNCs coming out of the insoluble fraction mask the translation elongation defect due to RNC aggregation.

If one considers a model in which Not5 partitions mRNAs out of solution when it binds ribosomes with a non‐optimal A site codon, why does this correlate with Not5‐dependent higher mRNA instability the more the mRNA has non‐optimal codons? It could be that mRNAs that aggregate as RNCs in the absence of Not5 are not as effectively turned over as mRNAs that are co‐translationally degraded or post‐translationally degraded in solution. In addition, based upon at least one example (S. Chen et al., 2023) (see below), we can propose that non‐optimal codons contribute by their impact on speed of translation, to targeting of mRNAs for instance to membranes, where they can be turned over by quality control mechanisms and this mRNA turnover is not related to deadenylation.

### 5.5 Ribosome pausing to increase membrane targeting

**FIGURE 6:**
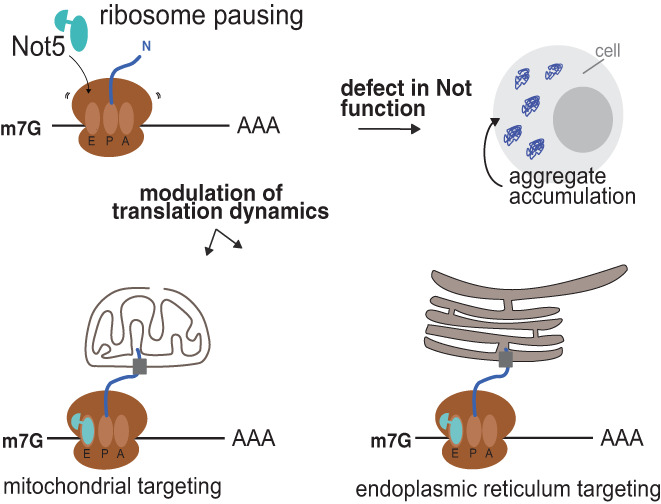
Ribosome pausing during translation is important for Not‐dependent co‐translational mRNA targeting. During translation the ribosome can slow down, for instance when it encounters non‐optimal codons, and upon prolonged pausing the E‐site can become empty such that the Ccr4‐Not complex via Not5 can interact with the ribosome. This Not5 binding enables co‐translational events such as mitochondrial or endoplasmic reticulum targeting of the nascent chain. In the absence of Not function, new inappropriately targeted or misfolded nascent chains can aggregate. Notably, mRNAs associated with membranes or in aggregates are likely to be insoluble, depending upon salt and detergent conditions.

The fact that Not proteins regulate mRNA solubilities and dynamics of co‐translational events as described above raises the question of what insoluble mRNAs are. Insoluble mRNAs could simply be RNCs that have aggregated because of nascent chain aggregation, or they could be mRNAs anchored at membranes. Finally, they could be mRNAs within functional condensates or even within RNA‐protein (RNP) condensates that are associated with membranes (Figure 6). Hence, to understand how the Not proteins impact dynamics of co‐translation events, it will be essential to characterize the nature of the insoluble mRNAs whose solubility is regulated by the Not proteins. A recent study focused on one mRNA, *MMF1* encoding a mitochondrial matrix protein, whose solubility decreased upon Not1 depletion but increased upon Not4 depletion. It could be determined that Not4 contributed to ribosome pausing itself, in turn permitting more effective co‐translational targeting to the mitochondria outer membrane. It was found that the ribosome pause site on *MMF1* had a non‐optimal A site codon. Ribosomes at this pause site can be expected to be targets for Not5 binding, and in turn Not5 binding might slow translation enough to promote co‐translational targeting (Figure 6). Mitochondrial mRNAs are enriched amongst mRNAs whose solubility is inversely modulated by Not1 and Not4 (Allen et al., 2023), and thus mitochondrial targeting may be an important focus of regulation by the Not proteins. How Not1 and Not4 would exert opposite effects for this is still to be determined.

Beyond mitochondria, membrane targeting via ribosome pausing is a possible mechanism by which the Not proteins can modulate solubility and dynamics of co‐translation events, including mRNA turnover. Recently new data has suggested that CNOT1 is required for targeting mRNAs to the ER and allowing translation of ER targeted mRNAs in human cells (Gillen, Giacomelli, et al., 2021) (Figure 6). Depletion of CNOT1 resulted in ER targeted mRNAs residing in the cytoplasm and not getting delivered to the ER. Interestingly, ribosome profiling shows that ER targeted mRNAs have ribosome protected fragments covering the first section of the mRNAs which coincides with the signal recognition motif (SRM), but ribosome occupancy plummets following this region suggesting a defect in targeting. Recent examination of collided ribosomes, disome sequencing, has revealed a build of disomes over SRMs (Arpat et al., 2020) and may indicate mechanistically how the Ccr4‐Not complex might be involved in ER targeting. A role of RNP condensates bearing a known Ccr4‐Not interacting protein Tis11 enabling translation in the vicinity of the ER has also been described (Ma & Mayr, 2018). It is unclear exactly how the Ccr4‐Not complex is participating in ER targeting and how this function acts in conjunction with the signal recognition particle (SRP). It is, however, tempting to speculate that ribosome stalling, in tandem or independently with the SRP, may trigger translocation of these stalled ribosome nascent chain complexes to the ER, where translation could resume.

### 5.6 Co‐translation quality control processes

In *S. cerevisiae*, mRNA stability according to codon optimality was initially investigated by modifying *PGK1* codon optimality (Hoekema et al., 1987) and then the relationship between mRNA stability and codon optimality was studied in depth using transcription shut‐off and an *rpb1‐1* mutant (Presnyak et al., 2015) or reporter constructs with different contents in optimal codons transcribed from a *GAL* promoter and transferring cells growing in galactose to glucose to follow mRNA decay (Buschauer et al., 2020; Presnyak et al., 2015). Greater instability of constructs with higher non‐optimal codon content was indicated to depend upon Dhh1 (Radhakrishnan et al., 2016) and then upon Not5 (Buschauer et al., 2020). These experiments were done under conditions where cells are switched from 24 to 37°C or from respiration to fermentation, and one unknown in these experiments is how a change in metabolism might play a role. Nevertheless, other experiments have also suggested that mRNAs with a higher non‐optimal codon content are more unstable, and this is also the case in mammalian cells (Hia et al., 2019; Q. Wu et al., 2019).

Using a fully reconstituted biochemical system with proteins from the fission yeast *Schizosaccharomyces pombe*, translation rates and deadenylation could be connected (Webster et al., 2018). Ccr4 was noted to be a general deadenylase working on all mRNAs because Pab1 is released from poly(A) tails in a Ccr4‐dependent manner. Caf1 on the other hand only trims poly(A) not bound by Pab1 and is thus a specialized deadenylase, required for the selective deadenylation of transcripts with lower rates of translation elongation and reduced Pab1 occupancy. In *S. cerevisiae*, transcripts with optimal codons were noted to have higher Pab1 occupancy, undergo slow poly(A) tail removal, and be not dependent on Caf1 for deadenylation. While these experiments establish an interesting connection between translation speed according to codon optimality, Pab1 occupancy and the deadenylating enzymes, they fail to establish a further link between codon optimality and mRNA turnover.

As mentioned above, recently it was shown that Not4 is important for ribosome pausing and co‐translational mitochondrial targeting of an overexpressed mitochondrial mRNA, and at the mitochondrial surface, the mRNA could then be turned over co‐translationally by the combined actions of No‐Go‐Decay (NGD) and autophagy (S. Chen et al., 2023). Such mechanisms may be more general, for instance if the Not proteins can tether mRNAs to membranes and/or granules where either the nascent protein will encounter its partner and then be further translated or if this does not occur the mRNA will be degraded by quality control mechanisms. If this is the case, then one can really think of the Ccr4‐Not complex as a quality control machine that contributes to the translation process and to define the fate of mRNAs according to the quality of the translation process.

### 5.7 Not4‐dependent ubiquitination

The key role of Not4‐dependent ubiquitination for this quality control role of the Ccr4‐Not complex is becoming increasingly apparent. As mentioned above, NAC was the first Not4 substrate identified, followed shortly thereafter by the ribosomal protein Rps7A, and a very limited number of other proteins such as Jhd2 (Huang et al., 2010; Mersman et al., 2009), Yap1 (Gulshan et al., 2012) and Srb10 (Cooper et al., 2012) in yeast. An interesting difference between NAC and Rps7A and the other early identified targets of ubiquitination by Not4, is that NAC and Rps7A show discrete ubiquitination and that the ubiquitinated proteins are stable, whilst other substrates are destabilized by Not4 ubiquitination. A third substrate of Not4 involved in the translation process was subsequently identified, first in flies (Z. Wu et al., 2018), but then confirmed in yeast (Allen et al., 2021). This is the Rli1/ABCE1 ATPase important for ribosome recycling after translation termination (Young et al., 2015), described also as a stable polyubiquitinated Not4 substrate. As will be discussed below, for all of these Not4 substrates associated with the ribosome, there is now evidence that their ubiquitination by Not4 plays important roles in co‐translational processes.

The function for NAC ubiquitination has only just emerged this year (S. Chen et al., 2023) despite it being the first identified Not4 substrate (Panasenko et al., 2006). The Egd1β NAC subunit binds nascent chains of mitochondrial proteins to target them to the Om14 mitochondrial outer membrane (MOM) protein co‐translationally (Lesnik et al., 2014). Its ubiquitination by Not4 in turn contributes to an integrated co‐translational quality control response occurring at the MOM, named Mito‐ENCAY, to limit overexpression of mitochondrial precursor proteins (S. Chen et al., 2023). Mito‐ENCAY describes a co‐translational quality control response for overexpressed nuclear‐encoded mRNAs encoding mitochondrial precursor proteins. It entails ribosome pausing to enable ribosome‐nascent chain complexes (RNCs) to be targeted and docked to the MOM, increased ribosome pausing at the MOM, ribosome‐quality control (RQC) as well as additional ubiquitination of components of the RNCs by Not4, followed by fission and clearance of fragmented mitochondrial vesicles carrying ubiquitinated RNCs by autophagy. Presumably, this NAC ubiquitination contributes to the overall ubiquitination of RNCs at the MOM that will be recognized by the autophagy machinery and targeted for degradation by autophagy. Caf130 known to anchor NAC to the Ccr4‐Not complex (J. Chen et al., 2001; Pillet et al., 2022) is important for this response but not for NAC ubiquitination by Not4, consistent with the fact that both Not4's E3 ligase domain and its interaction with Not1 are needed.

Ubiquitination of Rps7A by Not4 is important for Not5 presence in polysomes (Buschauer et al., 2020; Panasenko & Collart, 2012). Rps7A is only detected in ubiquitinated form in 80S or polysome fractions but not in 40S, meaning it is either only ubiquitinated when 40S are assembled with 60S or it is de‐ubiquitinated in 40S ribosomes. One model could be that it gets ubiquitinated after ribosome scanning at the start site when Not4 and Not5 are recruited to the fully assembled ribosomes. It has been proposed that Rps7A must be de‐ubiquitinated by Otu2 to enable translation re‐initiation (Ikeuchi et al., 2023; Takehara et al., 2021). Ubiquitination of Rps7A by Not4 regulates ribosome stalling and quality control responses (Allen et al., 2021; S. Chen et al., 2023; Panasenko & Collart, 2012). Indeed, Rps7A monoubiquitinated by Not4 can be further polyubiquitinated by the Hel2 ligase for ribosome quality control (RQC), if the RQC trigger complex (RQT, composed of Hel2, Slh1, Cue3, Rqt4) is defective (Ikeuchi et al., 2019). While this is not a physiological situation, this mechanism might be important for quality control responses such as Mito‐ENCay (S. Chen et al., 2023). Moreover, translation through specific stalling sequences (R12) is enabled if Rps7A is not ubiquitinated (Allen et al., 2021). A common theme in these quality control mechanisms that are affected by Rps7A ubiquitination is ribosome pausing, in turn likely to result in ribosome collisions.

In flies CNOT4 ubiquitinates the ribosome release ATPase ABEC1 (ortholog of yeast Rli1) upon mitochondrial damage, when translationally arrested respiratory chain mRNAs increase rapidly at the mitochondrial surface and co‐translational quality control factors are recruited to mitochondria. Together with broad ubiquitination of outer membrane proteins by the Parkin E3 ligase recruited in a PINK1‐dependent manner to damaged mitochondria, this recruits autophagy receptors to mitochondria to initiate mitophagy (Z. Wu et al., 2018). PINK1 with Tom20 helps to localize initially translationally repressed mRNAs to mitochondria leading to their de‐repression for co‐translational import of encoded proteins (Gehrke et al., 2015). In yeast, Rli1 is also ubiquitinated by Not4, and overexpression of Rli1, either wild type or with 16 mutated lysines, increases translation through an R12 stalling sequence in wild type cells but not in *not4Δ* or in a RING mutant (Allen et al., 2021). This suggests that overexpressed Rli1 works in combination with another target of Not4 to promote translation through R12. This hypothesis has yet to be tested. In any event, overexpression of Rli1 also inhibits Mito‐ENCay, that itself depends upon ribosome pausing (S. Chen et al., 2023).

Taken together these results indicate that Not4 ubiquitination of ribosome associated targets contributes to dynamics of co‐translation events, ribosome pausing being important both for co‐translation association of proteins and co‐translational targeting. Notably, ubiquitination of Rps7A appears to be at the intersection of re‐initiation and quality control. It remains to be determined whether ubiquitination of ribosome associated targets in mammalian cells is performed by CNOT4 alone, or CNOT4 together with RNF219, and similarly contributes to dynamics of co‐translation events?

## 6 TRANSLATION TERMINATION

There is not much information about a link between translation termination and the Ccr4‐Not complex. However, it is important to note that one of Not4's ubiquitination targets is Rli1 that recycles ribosomes. 5′P‐Seq data has indicated that ribosomes accumulate at the stop codon in the insoluble RNA pool. Upon depletion of Not1, Not4 or Not5, ribosomes accumulate at stop in the soluble RNA pool and for the insoluble RNA pool, the increase in pausing at stop is detectable only upon Not4 depletion (Allen et al., 2023). This suggests that the Not proteins might indeed impact translation termination at the step of ribosome release, maybe by changing solubility of mRNAs with ribosomes at the stop codon. Because Not4 seems to play a prominent role, it could be that the ubiquitination of Rli1 by Not4 is important; however, this requires investigation.

## 7 CONCLUSION AND PERSPECTIVES

It is now established that the vast majority of mRNAs require the Ccr4‐Not complex for their demise, and the accelerated delivery of this complex to mRNAs through a multitude of distinct mechanisms defines the mRNA profiles of the cell. This is no over statement, while transcription is responsible for the production of mRNAs, to achieve appropriate mRNA expression profiles within a cell the specific decay rate of each mRNA is equally important and controlled accordingly. However, deadenylation may not be the key rate limiting step leading to mRNA turnover described in many reviews and textbooks, and the essential role of the Ccr4‐Not complex for mRNA turnover is still not fully understood. Moreover, the role of the Ccr4‐Not complex goes well beyond just dictating decay rates of transcripts.

As covered in this review, it now transpires that the Ccr4‐Not complex is controlling translation at multiple levels, and this can operate independently of its deadenylation activity. Perhaps most transformative in recent years is the realization that this complex is surveying the progression of elongating ribosomes through the Not5/CNOT3 (yeast/mammalian) subunits interacting with the E site of the ribosome when the A site is empty (Absmeier et al., 2023; Buschauer et al., 2020). This enables the Ccr4‐Not complex to read an additional level of information superimposed upon the genetic code. The degenerate nature of the genetic code means that multiple codons can code the same amino acid. However, the speed at which these different codons are decoded by the ribosome can result in the ribosome having empty A and E sites, depending on several factors including, tRNA abundance and charging, tRNA modifications and competitive decoding environment, to name but a few. These factors can of course be adaptive in nature and allow monitoring of cellular conditions (Gingold et al., 2014).

This dynamic mechanism of sensing stalled elongation ribosomes allows the genome to store additional information at the codon level and uses this extra information to signal specific co‐translation events. At the basic level this information can be used to initiate degradation of the mRNAs that have poor codon optimality engaging the conventional decay pathways (Gillen, Giacomelli, et al., 2021). This mechanism is a widespread and major determinant to mRNA levels. However, this mechanism is clearly being employed to conduct more elaborate operations. Embedded information within the codon sequence changes ribosome elongation rates at specific places by directing, via the recruitment of the Ccr4‐Not complex, phase‐separation of these stalled ribosomes with nascent polypeptides emerging. This pausing during the elongation cycle appears critical for the production of functional protein products. Examples have now been documented where the Ccr4‐Not complex is required for co‐translation assembly of protein complexes (Figure 3b). It is unclear how widespread this phenomenon is, but it is tempting to extrapolate that this is occurring not just to allow correct protein complex assembly but also to allow proteins to fold correctly by pausing at specific regions to allow intermediate structures to form or to associate with a chaperone to facilitate folding. Differential codon usage has been shown to be critical for correct protein folding and production of functional proteins; however, the role of Ccr4‐Not complex in this process is still to be fully explored. It should also be mentioned that it is presently not clear whether it is the entire Ccr4‐Not complex (nine subunits in yeast, eight in mammals) that associates with the ribosome.

Finally, the Ccr4‐Not complex is also used to target delivery of proteins to the correct cellular location co‐translationally. Both trafficking mRNAs to the ER and mitochondria membranes appear to be key roles played by the Ccr4‐Not complex (Figure 6). These compartments are relatively easy to identify both experimentally and bioinformatically, but whether the Ccr4‐Not complex is more globally involved in delivering proteins to their correct cellular location is currently unclear.

## AUTHOR CONTRIBUTIONS

**Martine Collart:** Conceptualization (equal); data curation (equal); formal analysis (equal); funding acquisition (equal); writing – original draft (equal); writing – review and editing (equal). **Léna Audebert:** Writing – original draft (supporting); writing – review and editing (supporting). **Martin Bushell:** Conceptualization (equal); data curation (equal); formal analysis (equal); funding acquisition (equal); writing – original draft (equal); writing – review and editing (equal).

## FUNDING INFORMATION

This work was supported by grant 310030‐207420 from the Swiss National Science Foundation awarded to M.A.C., grants A17196 and A31287 from Cancer Research UK to the CRUK Beatson Institute and A29252 to the Bushell lab.

## CONFLICT OF INTEREST STATEMENT

The authors declare no conflicts of interest.

## RELATED WIREs ARTICLES

Novel roles of the CCR4‐NOT complex

The Ccr4‐Not complex is a key regulator of eukaryotic gene expression

Ribosome dynamics and mRNA turnover, a complex relationship under constant cellular scrutiny

## Data Availability

Data sharing is not applicable to this article as no new data were created or analyzed in this study.
